# Comparing Health Outcomes of Privileged US Citizens With Those of Average Residents of Other Developed Countries

**DOI:** 10.1001/jamainternmed.2020.7484

**Published:** 2020-12-28

**Authors:** Ezekiel J. Emanuel, Emily Gudbranson, Jessica Van Parys, Mette Gørtz, Jon Helgeland, Jonathan Skinner

**Affiliations:** 1 Department of Medial Ethics and Health Policy, Perelman School of Medicine, University of Pennsylvania, Philadelphia; 2 Department of Healthcare Management, The Wharton School, University of Pennsylvania, Philadelphia; 3 School of Medicine, Yale University, New Haven, Connecticut; 4 Department of Economics and Accounting, Hunter College, CUNY, New York, New York; 5 Department of Economics, University of Copenhagen, Copenhagen, Denmark; 6 Division for Health Services, Norwegian Institute of Public Health, Oslo, Norway; 7 Department of Economics, Dartmouth College, Hanover, New Hampshire; 8 Dartmouth Institute for Health Policy and Clinical Practice, Geisel School of Medicine, Lebanon, New Hampshire

## Abstract

**Importance:**

The average health outcomes in the US are not as good as the average health outcomes in other developed countries. However, whether high-income US citizens have better health outcomes than average individuals in other developed countries is unknown.

**Objective:**

To assess whether the health outcomes of White US citizens living in the 1% and 5% richest counties (hereafter referred to as privileged White US citizens) are better than the health outcomes of average residents in other developed countries.

**Design, Setting, and Participants:**

This comparative effectiveness study, conducted from January 1, 2013, to December 31, 2015, identified White US citizens living in the 1% (n = 32) and 5% (n = 157) highest-income counties in the US and measured the following 6 health outcomes associated with health care interventions: infant and maternal mortality, colon and breast cancer, childhood acute lymphocytic leukemia, and acute myocardial infarction. The study used Organisation for Economic Co-operation and Development data, CONCORD-3 cancer data, and Medicare data to compare their outcomes with all residents in 12 other developed countries: Australia, Austria, Canada, Denmark, Finland, France, Germany, Japan, the Netherlands, Norway, Sweden, and Switzerland. Statistical analysis took place from July 25, 2017, to August 29, 2020.

**Main Outcomes and Measures:**

Infant mortality; maternal mortality; 5-year survival of patients with colon cancer, breast cancer, or childhood acute lymphocytic leukemia; and 30-day age-standardized case fatality after acute myocardial infarction.

**Results:**

The infant mortality rate among White US citizens in the 5% highest-income counties was 4.01 per 1000, and the maternal mortality rate among White US citizens in the 5% highest-income counties was 10.85 per 100 000, both higher than the mean rates for any of the 12 comparison countries. (The infant mortality rate for the top 1% counties was 3.54 per 1000, and the maternal mortality rate was 10.05 per 100 000.) The 5-year survival rate for White US citizens in the 5% highest-income counties was 67.2% (95% CI, 66.7%-67.7%) for colon cancer, higher than that of average US citizens (64.9% [95% CI, 64.7%-65.1%]) and average citizens in 6 countries, comparable with that of average citizens in 4 countries, and lower than that of average citizens for 2 countries. The 5-year survival rate for breast cancer among White US women in the 5% highest-income US counties was 92.0% (95% CI, 91.6%-92.4%), higher than in all 12 comparison countries. The 5-year survival rate for White children with acute lymphocytic leukemia in the 5% highest-income US counties was 92.6% (95% CI, 90.7%-94.2%), exceeding the mean survival rate for only 1 country and comparable with the mean survival rates in 11 countries. The adjusted 30-day acute myocardial infarction case-fatality rate for White US citizens in the 5% highest-income US counties was 8% below the rate for all US citizens and was 5% below the rate for all US citizens in the 1% highest-income US counties; these estimates were similar to the median outcome of other high-income countries.

**Conclusions and Relevance:**

This study suggests that privileged White US citizens have better health outcomes than average US citizens for 6 health outcomes but often fare worse than the mean measure of health outcomes of 12 other developed countries. These findings imply that even if all US citizens experienced the same health outcomes enjoyed by privileged White US citizens, US health indicators would still lag behind those in many other countries.

## Key Points

**Question:**

Are the health outcomes of White US citizens living in the 1% and 5% richest counties better than the health outcomes of average residents in other developed countries?

**Findings:**

In this comparative effectiveness study of 6 health outcomes, White US citizens in the 1% and 5% highest-income counties obtained better health outcomes than average US citizens but had worse outcomes for infant and maternal mortality, colon cancer, childhood acute lymphocytic leukemia, and acute myocardial infarction compared with average citizens of other developed countries.

**Meaning:**

For 6 health outcomes, the health outcomes of White US citizens living in the 1% and 5% richest counties are better than those of average US citizens but are not consistently better than those of average residents in many other developed countries, suggesting that in the US, even if everyone achieved the health outcomes of White US citizens living in the 1% and 5% richest counties, health indicators would still lag behind those in many other countries.

## Teaser

This comparative effectiveness study assesses whether the health outcomes of White US citizens living in the 1% and 5% richest counties are better than the health outcomes of average residents in other developed countries.

## Introduction

The US health care system appears to underperform on nearly every metric. The US spends more than $3.5 trillion per year on health care, 25% more per capita than the next highest-spending country.^1^ However, compared with other countries, the US performs poorly on process, outcome, and patient experience metrics, as well as life expectancy. Compared with countries tracked by the Commonwealth Fund, the US ranks behind every country on causes of preventable mortality that could have been addressed by health system interventions.^2^

Despite these well-known data, US politicians, the public, and even physicians seem complacent, often proclaiming that the United States has “the best health care system in the world.”^3,4,5^ As a recent poll showed, “nearly 80 percent of Americans reflect positively on the health care they personally receive.”^6^ Similarly, in a recent Gallup poll, approximately two-thirds of US citizens said they were “completely or mostly satisfied with the US healthcare system.”^7^ Why is the disconnect between the health care system’s performance and our personal perception of quality so pervasive?

Privileged US citizens—including thought and physician leaders—may tolerate this underperformance as applying to “others,” dismissing comparisons as mean values that do not reflect the quality of their own personal care.^8^ Privileged US citizens believe that their social connections and financial resources allow them to choose the best physicians and hospitals for their own care, thereby ensuring excellent health outcomes.^9^ One study showed that the wealthiest quintile receive 43% more health care than the poorest quintile and 23% more than middle-income US citizens.^10^ Privileged US citizens may believe that their resources ensure that they receive the world’s best health care, even if less advantaged US citizens cannot.

To our knowledge, no study has compared the health outcomes of privileged US citizens with those of average citizen*s* in other countries. Thus, to evaluate whether privileged US citizens truly have the best health outcomes in the world, we compared the health outcomes of White US citizens in the 1% and 5% highest-income US counties (hereafter referred to as privileged White US citizens) with those of average individuals in other developed countries. This comparison allows us to quantify the potential limits to erasing pervasive inequality in US health care by race/ethnicity and income^11,12,13^; how well would the US rank against comparison countries if every citizen in the US experienced health outcomes commensurate with privileged US citizens? We examined the following 6 health outcomes that are associated with the timeliness and quality of health care services: infant mortality; maternal mortality; 5-year survival of patients with colon cancer, breast cancer, and childhood acute lymphocytic leukemia (ALL); and 30-day case-fatality rates after acute myocardial infarction (AMI).

## Methods

In this comparative effectiveness study, conducted from January 1, 2013, to December 31, 2015, we identified the top 1% and 5% highest-income counties for White US citizens using median family income from the 2015 Census Bureau’s Small Area Income and Poverty Estimates.^14^ Statistical analysis took place from July 25, 2017, to August 29, 2020. A total of 157 of 3142 counties were included for analysis of the 5% highest-income counties, with 32 representing the 1% highest-income counties. They are located in the District of Columbia and 35 states, ranging from Montgomery County, Maryland, to St Johns County, Florida (full list in eAppendix 2 in the Supplement). We identified 12 comparison countries—Australia, Austria, Canada, Denmark, Finland, France, Germany, Japan, the Netherlands, Norway, Sweden, and Switzerland—that span 4 continents. We obtained mean annual income, per capita health expenditures, and life expectancy variables from the Organisation for Economic Co-operation and Development (OECD). The analysis of US AMI data aggregated to the county level was approved by the Dartmouth Committee on the Protection of Human Subjects. Because this study used publicly available, preexisting, aggregate data, it was deemed exempt from institutional review board review by the University of Pennsylvania Institutional Review Board.

### Infant Mortality

Infant mortality from comparison countries was calculated using 2014-2015 OECD data. For the 1% and 5% highest-income counties in the US, we calculated the infant mortality rate per 1000 live births for each county using the Centers for Disease Control and Prevention Underlying Cause of Death, 1999-2015, data set. We identified White non-Hispanic infant deaths (<1 year) at the county level from 2011 to 2015, generating a 5-year rolling mean of the infant mortality rate per 1000 for each of the US counties in the top 1% and 5% of counties by income.

### Maternal Mortality

County-specific data on maternal mortality were obtained from the National Center for Health Statistics Vital Statistics Mortality–All County (microdata) data files. We identified White non-Hispanic maternal deaths at the county level from 2011 to 2015, generating 5-year rolling mean values of the maternal mortality rate per 100 000 for each of the top 1% and 5% highest-income US counties.

### Cancer Survival

Five-year cancer survival rates for adult breast and colon cancer and for childhood ALL for comparison countries were taken from the CONCORD-3, which uses population-based cancer registries and standardized methods.^15^ To ensure comparability between the US counties and foreign countries, we used the CONCORD-3 methods to obtain 5-year survival for the 157 highest-income counties in the US. To obtain county-specific data on colon cancer, breast cancer, and childhood ALL, we used the North American Association of Central Cancer Registries Cancer in North America Deluxe database. We aggregated 5-year cause-specific survival, relative survival, and net (Pohar Perme estimator) survival, both overall and stratified by race/ethnicity. Because of confidentiality concerns, not all states allowed access to their county-level data, but we were able to obtain data from counties in 34 states. Similarly, low incidence and confidentiality concerns precluded analyses of the top 1% of counties.

### AMI Mortality

We used 2 types of data to examine differences in AMI mortality across countries. Our primary data source was the OECD 30-day linked case fatality rates after AMI for patients 45 years or older in the US and 10 other high-income countries that reported data in both years: Canada, Denmark, Finland, Israel, Italy, New Zealand, Norway, Spain, Sweden, and the United Kingdom.^16^ We used 2013-2014 OECD measures because these were the most recently available for the US.

We supplemented the OECD data with more detailed analysis of patient-level data from the US, Norway, and Denmark. First, to estimate AMI case-fatality rates for the highest-income US counties relative to the rest of the country, we required the use of the 100% Medicare fee-for-service claims data (from January 2013 to September 2015) at the county level, with a subset of individuals identified as White, matched by zip code to the highest-income counties. We identified inpatient episodes for which AMI was both the primary admitting diagnosis and the patient’s first AMI hospitalization using *International Classification of Diseases, Ninth Revision* codes 410.xx (except 410.x2). We applied the estimated case-fatality relative risk for the wealthy county sample relative to all Medicare enrollees to the OECD data on individuals 45 years or older to create an imputed measure of case-fatality rates among high-income US citizens 45 years or older.

Second, there were concerns about potential biases in the US OECD data because the data were limited to a subset of states and were based on the Healthcare Utilization and Cost Project, which excludes deaths occurring outside the hospital. Consequently, we performed direct comparisons of 30-day case-fatality rates from US Medicare data (for those ≥65 years) with age-adjusted and sex-adjusted case-fatality rates from similar 100% samples in Norway and Denmark. In all 3 countries, case-fatality rates included those who died outside the hospital. These individual analyses were then compared with the OECD data to assess potential biases directly (eAppendix 1 in the Supplement). Finally, as a sensitivity analysis, we estimated case-fatality rates for the top (and bottom) 5% of zip codes by median income in the US.

## Results

The 157 richest US counties have a median household income of approximately $84 000, higher than the mean annual income in Switzerland (US $62 495), Norway (US $51 663), and the other comparison countries (eTable 1 in the Supplement). Per capita health care expenditures in the highest-income US counties are not available, but the US had substantially higher per capita spending in 2015 than any other country—$9491 per capita, compared with US $7570 in Switzerland and US $6239 in Norway (eTable 1 in the Supplement).

### Infant Mortality

The infant mortality rate among White US citizens in the 1% highest income counties is 3.54 per 1000 live births, while the 5% highest-income counties have an infant mortality rate of 4.01 per 1000 live births—higher than in all 12 comparison countries (eTable 2 in the Supplement). Among all US citizens, the infant mortality rate is 5.90 deaths per 1000 live births (eTable 2 in the Supplement). Among comparison countries, the infant mortality rate is lowest in Finland, at 1.70 per 1000 live births, and highest in Canada, with 4.70 per 1000 live births. Only 2 of the top 157 highest-income counties in the US have White infant mortality rates below that of Norway, and none have rates lower than Finland (eTable 2 in the Supplement).

### Maternal Mortality

The maternal mortality rate among White US citizens in the 1% and 5% highest-income counties is higher than in any other comparison country (eTable 3 in the Supplement). The maternal mortality rate is 26.40 per 100 000 live births among all US women. Among White US women, the maternal mortality rate is 10.05 per 100 000 births in the 1% highest-income counties and 10.85 per 100 000 births in the 5% highest-income counties (eTable 3 in the Supplement). Even in California, which has implemented a major initiative to reduce maternal mortality since 2006, the mortality rate for White mothers is 7.3 per 100 000 live births.^17^ Outside of the US, the worst-performing countries are Canada, with 6.00 maternal deaths per 100 000 births, and France, with 5.10 maternal deaths per 100 000 births.

### Cancer Survival

The 5-year survival rate for colon cancer among all US citizens is 64.9% (95% CI, 64.7%-65.1%). For White US citizens in the 5% highest-income US counties, the survival rate is 67.2% (95% CI, 66.7%-67.7%). This survival rate was higher than that in 7 other countries but comparable to rates for average citizens in Canada, Japan, Norway, and Switzerland. However, average Australian citizens have a higher survival rate, at 70.7% (95% CI, 70.1%-71.2%), than privileged White US citizens (eTable 4 in the Supplement).

The 5-year survival rate for breast cancer among White US women in the 5% highest-income US counties is 92.0% (95% CI, 91.6%-92.4%), higher than that for all US women with breast cancer (90.2% [95% CI, 90.1%-90.4%]) (eTable 4 in the Supplement). Breast cancer survival is higher in the US than for average citizens in all the comparator countries; the countries with the next highest breast cancer survival rates among average citizens are Australia (89.5% [95% CI, 89.1%-90.0%]), Japan (89.4% [95% CI, 88.9%-89.9%]), and Sweden (88.8% [95% CI, 88.2%-89.4%]) (eTable 4 in the Supplement).

The 5-year survival rate for ALL among average US children is 89.5% (95% CI, 88.8%-90.3%). The 5-year survival rate for White children in the 5% highest-income US counties is 92.6% (95% CI, 90.7%-94.2%) (eTable 4 in the Supplement). The survival rate for White children in the 5% highest-income US counties is higher than the survival rate in only 1 country—Norway—and is comparable in almost all other countries. Average children in Denmark (94.0% [95% CI, 90.1%-97.9%]) and Finland (95.2% [95% CI, 91.5%-98.9%]) have higher 5-year survival rates than White children in the 5% highest-income US counties, whose rate is similar to that of average children in Canada (92.6% [95% CI, 90.7%-94.6%]) (eTable 4 in the Supplement).

### Acute Myocardial Infarction

We began with the individual-level analysis of individuals 65 years or older in the US (with the top 1% of counties by income, the top 5% of counties by income, and the entire US), Denmark, and Norway (eTable 5 and eAppendix 1 in the Supplement). The age-standardized and sex-standardized 30-day case-fatality rate for AMI among White US citizens 65 years or older in the wealthiest 1% of counties is 12.7%, somewhat above the 12.4% case-fatality rate for the top 5% of counties by income. These rates are significantly lower than for the general US population (13.4%) but substantially higher than in Norway (10.2%) and Denmark (10.7%).

As a sensitivity analysis, we considered case-fatality rates for White individuals in the Medicare program aged 65 years or older living in the top 5% of zip codes by income; for these patients, whose mean zip code income is $117 401, the case-fatality rate is 12.0%, which is less than the case-fatality rate for the 5% of counties with the highest income but, again, greater than in Norway and Denmark. For people in the lowest 5% of zip code income, the case-fatality rate is 14.7%, well above the US national mean.

From the OECD data, the 30-day case fatality rate for average US citizens is 8.8 per 100 patients with AMI (eFigure in the Supplement), lower than Finland (8.9 per 100 patients with AMI) and the United Kingdom (9.2 per 100 patients with AMI), similar to Israel, and higher than 7 other countries. We used our finding from the Medicare claims data on mortality in high-income counties to adjust the OECD data. These data estimate that the OECD case-fatality rate is 8.1% for the top 5% of counties and 8.4% for the top 1% of counties; these 2 estimates for health outcomes among privileged White US citizens bracket the median measure of comparison countries (eFigure in the Supplement). An alternative approach adjusting for the potential underreporting of US out-of-hospital deaths suggests that adjusted US mortality rates are worse than the median OECD country, whether county-level or zip code–level income measures are used (eAppendix 1 in the Supplement).

## Discussion

The health outcomes of privileged White US citizens for 6 health outcomes are better than those for average US citizens; however, the health outcomes of privileged White US citizens for infant mortality, maternal mortality, and AMI are not consistently better than the outcomes of average residents in many other developed countries. For health conditions for which the outcomes are associated with the quality of health care, privileged US citizens—those who have high incomes and are White—do not always experience the best outcomes. Four points need emphasizing.

First, being well-off and White in the United States is associated with better health outcomes than those experienced by average US citizens.^18,19,20^ Well-off White US citizens have lower rates of infant and maternal mortality, increased 5-year cancer survival, and lower 30-day case fatality rates for AMI (conditional on reaching the hospital) compared with average US citizens. In general, within the US, social and economic capital is able to “buy” more health care services and better health outcomes for conditions that may be improved by medical interventions. This is consistent with the well-established finding that being well-off in the US and other countries is associated with longer life expectancy and better survival for certain health outcomes.^16,21^

However, being a White US citizen living in the 1% or 5% highest-income counties does not guarantee the world’s best health outcomes; in general, the outcomes for these individuals are no better than for average citizens in many other developed countries, and for infant, maternal, and AMI mortality, privileged White US citizens often fare worse. The pattern with cancer is more complicated. Privileged White US citizens appear to have the best outcome in the world for breast cancer. That outcome is very likely due to the high rate of mammogram screening in the US, which is associated with higher rates of diagnosis of small cancers.^22,23^ However, if undetected, most of these small cancers would not have progressed to large cancers and caused death. Consequently, there is a high 5-year survival rate but not a lower overall breast cancer mortality rate because mammography does not increase detection of larger tumors.^18,19,24^ In the case of colon cancer, privileged White US citizens had better survival than average citizens in most of the comparator countries; for childhood ALL, survival rates were similar across countries.

Third, many US citizens equate high-quality care with freedom of choice. They believe that having choice will engender better care, reflected by their higher satisfaction and increased access to services compared with individuals living in low-income countries.^10^ This study suggests that this belief may be true in a relative sense, in that wealth can improve the outcomes for some conditions compared with lower-income US citizens, but not in an absolute sense, as wealth does not guarantee the world’s best outcomes. The improvements produced by choice can be small, as in breast cancer; in other cases, such as for AMI, a patient may not even be able to exercise much choice because they are taken quickly to whichever hospital is nearby. Thus, choice may not be sufficient to ensure the best outcomes.

Fourth, even if the dramatic and pervasive inequalities in the provision of US health care across race/ethnicity and socioeconomic status were resolved, so that every US citizen experienced health outcomes consistent with those of privileged US citizens, the US would still not rank among the best of comparison countries.^11,12,13^ This finding makes it critically important to ask why well-off White US citizens do not have measurably better outcomes—and sometimes have worse outcomes—than average people in other developed countries. Our results suggest—but do not prove—that health outcomes depend on the system of care, rather than the performance of individual physicians or hospitals. For example, Chen et al^25^ found that the US lagged far behind other countries in infant and maternal mortality primarily because of adverse events, such as respiratory disease and accidents, occurring during the postneonatal period, well after the mother and baby have left the hospital.

Similarly, research indicates that harmful adverse events that qualify as malpractice are not the result of bad actions by a single physician or nurse but rather are caused by substandard processes and organization of care.^26^ Good care is less likely to be a matter of any one outstanding physician, and more the result of excellent systems of care. It is not an individual physician, for example, who “saves” a patient with AMI, but rather the coordinated response by emergency medical technicians, emergency department physicians, specialists trained in percutaneous cardiac interventions, and nurses and other clinicians in coronary care units. Similarly, excellent care for colon cancer depends on surgeons, medical oncologists, pharmacists, infusion nurses, and many other health care professionals in both the acute and postacute settings.^27^

Furthermore, avoiding hospital-acquired infections and other mistakes while being treated for these conditions does not depend on the care of a single physician. Therefore, choosing a concierge cardiologist or a hospital ranked highly by *U.S. News & World Report* may ensure prompt service and personalized attention, which have value, but it does not ensure the world’s best clinicians at each stage of care, at whatever facility is providing care, and does not ensure the best outcomes. A well-off US citizen cannot “buy out” of the uneven quality of care provided by the US health care system. To ensure the world’s best health outcomes requires improving care systematically, for all people at all facilities.

### Limitations

This study has several limitations. First, these results might not be generalizable. We compared the results for 6 health outcomes, which may not represent a complete picture of all health outcomes, nor of a health care system’s entire performance. Second, we are measuring health outcomes for high-income counties, rather than high-income individuals, which could lead to bias given that some low-income households reside in high-income counties. However, even using the top 1% of counties or recalculating (for the AMI data) for the top 5% of zip code income yielded largely similar results, although we recognize that much less is known about health outcomes for people at the top of income distribution.

Third, while most people receive health care near where they reside, some health care services, especially for cancer or AMI, might not be local. Thus, obtaining data from the 5% richest counties might not reflect the actual experiences of the residents. Fourth, we measure mortality and not quality of life. Patients who survive an AMI might have severe congestive heart failure that compromises their quality of life. Similarly, children who survive ALL might have serious cognitive effects or other complications of treatment.

Fifth, for all the conditions studied, health care is not the only factor associated with the outcome. Behavioral factors, such as obesity, diet, and sedentary lifestyle; environmental factors; and genetic factors are all associated with health outcomes and are difficult to compare across countries. By focusing on outcomes directly after common medical treatment, however, we have attempted to minimize the importance of these additional factors.

Sixth, countries might calculate health outcomes in slightly different ways that do not permit accurate comparisons. This is not true for the cancer outcomes, for which the results are reported based on standardized methods used in CONCORD-3, or for the AMI results with Denmark and Norway. Thus, it seems unlikely that the performance by the privileged White US citizens across these health conditions can be explained solely by differences in how outcomes were calculated.

## Conclusions

Compared with average citizens in many other developed countries, well-off White US citizens have worse outcomes in infant and maternal mortality and AMI. Privileged White US citizens do obtain better health outcomes than average US citizens for 6 health conditions, while low-income US citizens have much worse outcomes. However, being able to use social and financial capital in the US to buy the best health care is not necessarily associated with the world’s best health outcomes.

## Notes

Supplement. **eTable 1.** National Characteristics by Country **eTable 2.** Comparison of Infant Mortality Rates **eTable 3.** Comparison of Maternal Mortality Rates **eTable 4.** Comparison of 5-Year Survival Rates for Breast and Colon Cancer and Childhood ALL **eTable 5.** Comparison of 30-Day Mortality After Acute Myocardial Infarction **eFigure.** Adjusted Case Fatality Rate for Comparison Countries and the US for Overall, Top 1% and Top 5% Counties by Income, 2013-14 **eAppendix 1.** Statistical Appendix **eAppendix 2.** List of 157 Highest-Incomes Counties in the US
