# Supplementary material for: Comparing Health Outcomes of Privileged US Citizens With Those of Average Residents of Other Developed Countries
Source: JAMA Intern Med. 2020 Dec 28;181(3):1–6. doi: 10.1001/jamainternmed.2020.7484 (PMC7770612; doi:10.1001/jamainternmed.2020.7484)
Supplement: Supplement. — eTable 1. National Characteristics by Country eTable 2. Comparison of Infant Mortality Rates eTable 3. Comparison of Maternal Mortality Rates eTable 4. Comparison of 5-Year Survival Rates for Breast and Colon Cancer and Childhood ALL eTable 5. Comparison of 30-Day Mortality After Acute Myocardial Infarction eFigure. Adjusted Case Fatality Rate for Comparison Countries and the US for Overall, Top 1% and Top 5% Counties by Income, 2013-14 eAppendix 1. Statistical Appendix eAppendix 2. List of 157 Highest-Incomes Counties in the US [file jamainternmed-e207484-s001.pdf]

Supplementary Online Content

Emanuel EJ, Gudbranson E, Van Parys J, Gørtz M, Helgeland J, Skinner J. Comparing health outcomes of privileged US citizens with those of average residents of other developed countries. *JAMA Intern Med.* Published online December 28, 2020. doi:10.1001/jamainternmed.2020.7484

eTable 1. National Characteristics by Country
eTable 2. Comparison of Infant Mortality Rates
eTable 3. Comparison of Maternal Mortality Rates
eTable 4. Comparison of 5-Year Survival Rates for Breast and Colon Cancer and Childhood ALL
eTable 5. Comparison of 30-Day Mortality After Acute Myocardial Infarction
eFigure. Adjusted Case Fatality Rate for Comparison Countries and the US for Overall, Top 1% and Top 5% Counties by Income, 2013-14
eAppendix 1. Statistical Appendix
eAppendix 2. List of 157 Highest-Incomes Counties in the US

This supplementary material has been provided by the authors to give readers additional information about their work.

© 2020 Emanuel EJ et al. *JAMA Internal Medicine*.

eTable 1. National Characteristics by Country

Group	Country	Average Annual Wages PPP (2015)*	Health Expenditures per Capita, USD (2015)+	Life Expectancy (2015)
USA	Whites in 1% Richest Counties	$100,524	NA	NA
	Whites in 5% Richest Counties	$83,639	NA	NA
	All	$60,692	$9,491	78.7
Comparison Countries	Australia	$49,563	$4414	82.5
	Austria	$49,965	$5159	81.7
	Canada	$47,673	$4633	81.9
	Denmark	$51,126	$5001	80.8
	Finland	$42,885	$4099	81.6
	France	$42,731	$4657	82.4
	Germany	$46,409	$5297	80.7
	Japan	$40,004	$4428	83.9
	Netherlands	$53,171	$5148	81.6
	Norway	$51.663	$6239	82.4
	Sweden	$41,467	$5272	82.3
	Switzerland	$62,495	$7570	83.0

All data from OECD
** Average Annual Wages (PPP) from*

https://stats.oecd.org/Index.aspx?DataSetCode=AV_AN_WAGE

+ Health Expenditures per capita from OECD (2019), Health spending (indicator). doi: 10.1787/8643de7e-en (Accessed on 03 April 2019)

© 2020 Emanuel EJ et al. *JAMA Internal Medicine*.

eTable 2. Comparison of Infant Mortality Rates

Group	Country	Infant Mortality (deaths / 1,000 live births), 2015	Number of Top 5% Richest USA Counties Below the Infant Mortality Rate of the Comparison Country
USA	Whites in 1% Richest Counties	3.54	NA
	Whites in 5% Richest Counties	4.01	NA
	Overall	5.90	NA
Comparison Countries	Australia	3.20	17
	Austria	3.10	14
	Canada	4.70	139
	Denmark	3.70	38
	Finland	1.70	0
	France	3.70	38
	Germany	3.30	20
	Japan	1.90	0
	Netherlands	3.30	20
	Norway	2.30	2
	Sweden	2.50	7
	Switzerland	3.90	115

© 2020 Emanuel EJ et al. *JAMA Internal Medicine*.

eTable 3. Comparison of Maternal Mortality Rates

Group	Country	Maternal Mortality (deaths / 100,000 births), 2015
USA	Whites in 1% Richest Counties	10.05
	Whites in 5% Richest Counties	10.85
	Overall	26.40
Comparison Countries	Australia	2.60
	Austria	4.70
	Canada	6.00
	Denmark	0.00
	Finland	3.60
	France	5.10
	Germany	3.30
	Japan	4.40
	Netherlands	3.50
	Norway	0.00
	Sweden	0.90
	Switzerland	2.40

© 2020 Emanuel EJ et al. *JAMA Internal Medicine*.

eTable 4. Comparison of 5-Year Survival Rates for Breast and Colon Cancer and Childhood ALL

Group	Country	Breast Cancer	Colon Cancer	Childhood Acute Lymphocytic Leukemia (ALL)
USA	Whites in 5% Richest Counties	92.0% (91.7-92.3)	67.2% (66.7-67.7)	92.6% (90.7-94.2)
	Overall	90.2% (90.1-90.4)	64.9% (64.7-65.1)	89.5% (88.8-90.3)
Comparison Countries	Australia	89.5% (89.1-90.0)	70.7% (70.1-71.2)	90.7% (88.3-93.1)
	Austria	84.8% (84.1-85.5)	63.7% (62.7-64.7)	NA
	Canada	88.2% (87.8-88.6)	67.0% (66.5-67.5)	92.6% (90.7-94.6)
	Denmark	86.1% (85.4-86.9)	61.6% (60.6-62.7)	94.0% (90.1-97.9)
	Finland	88.5% (87.7-89.3)	64.9% (63.7-66.2)	95.2% (91.5-98.9)
	France	86.7% (85.5-88.0)	63.7% (62.2-65.3)	88.6% (86.5-90.8)
	Germany	86.0% (85.7-86.4)	64.8% (64.3-65.3)	91.1% (87.4-94.8)
	Japan	89.4% (88.9-89.9)	67.8% (67.3-68.4)	87.6% (84.2-91.0)
	Netherlands	86.6%	63.1%	90.4%

© 2020 Emanuel EJ et al. *JAMA Internal Medicine*.

		(86.1-87.1)	(62.5-63.7)	(87.5-93.3)
	Norway	87.7%<br>(86.6-88.8)	66.7%<br>(65.6-67.8)	83.0%<br>(76.5-89.5)
	Sweden	88.8%<br>(88.2-89.4)	64.9%<br>(64.0-65.8)	89.0%<br>(84.6-93.3)
	Switzerland	86.2%<br>(85.1-87.3)	67.3%<br>(65.7-68.9)	90.3%<br>(86.1-94.5)

:

© 2020 Emanuel EJ et al. *JAMA Internal Medicine*.

eTable 5. Comparison of 30-Day Mortality After Acute Myocardial Infarction

**Country**	**(Deaths / 100 AMI hospitalizations)**					
	**Overall**	**65-69**	**70-74**	**75-79**	**80-84**	**85+**
USA Whites in 1% Richest Counties (N = 10,229)	12.76	6.06	7.47	9.98	13.22	23.30
USA Whites in 5% Richest Counties (N = 46,382)	12.40	6.02	7.47	10.33	13.55	22.46
USA—Overall (N = 391,978)	13.45	6.73	8.62	11.01	14.59	23.34
Denmark (N = 15,567)	10.73	3.85	5.82	8.94	12.12	21.56
Norway (N = 18,564)	10.18	3.38	4.80	8.52	11.44	21.51

U.S. data is for 2013-15 in the fee-for-service Medicare population age 65+. Denmark data is for 2012-14, and Norway for 2013-15, both covering the entire population age 65+. Age-specific rates (and overall rates) are weighted by the sex distribution (and the age-sex distribution) for the Medicare U.S. population.

© 2020 Emanuel EJ et al. *JAMA Internal Medicine*.

eFigure. Adjusted Case Fatality Rate for Comparison Countries and the US for Overall, Top 1% and Top 5% Counties by Income, 2013-14

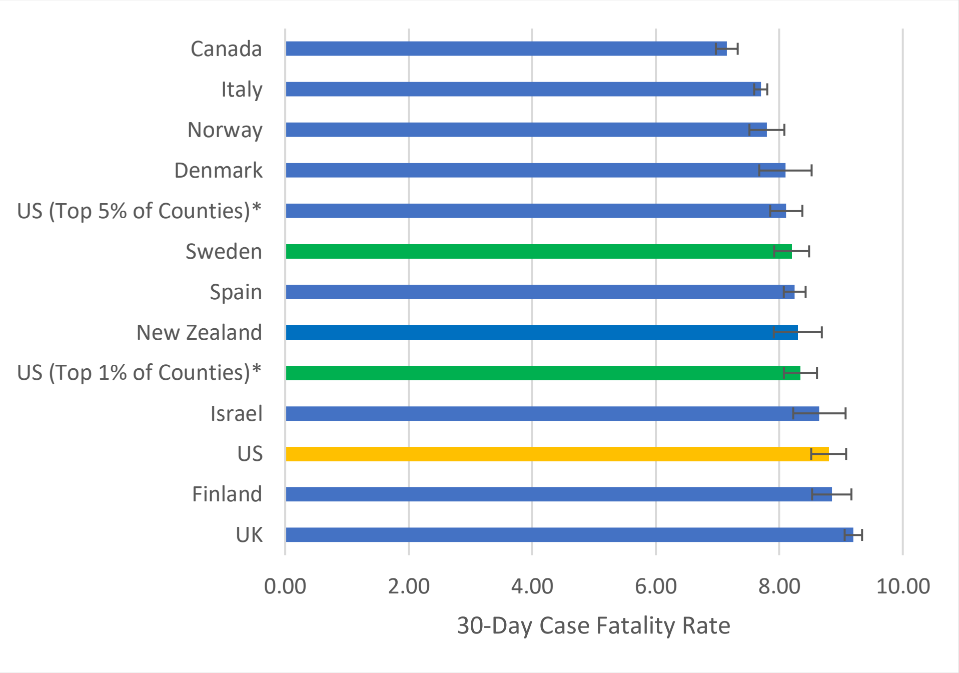

Notes: Data based on OECD health indicators for linked 30-day mortality, 2013-14. *The adjustments to US data by income group are made using the Medicare claims data (2013-15); see text and eAppendix 1 for details.

© 2020 Emanuel EJ et al. *JAMA Internal Medicine*.

eAppendix 1. Statistical Appendix

1. Micro-Level Data Analysis for Acute Myocardial Infarction

This appendix focuses on the data analysis for acute myocardial infarction (AMI). We first consider the micro-level analysis for the 65+ population in three countries with high-quality data from the U.S., Denmark, and Norway, and then the OECD data for ages 45+.

1. Micro-level claims data for the U.S., Denmark, and Norway

For the U.S. data, AMI was both the primary admitting diagnosis and the patient's first AMI hospitalization using ICD-9 codes 410.xx [except 410.x2]. The Master Beneficiary Summary File is used to identify dates of death for patients in this sample. We calculate the shares of patients by age group (65-69, 70-74, 75-79, 80-84, 85+) and sex who died within 30-days of their first AMI hospitalization observed during 2013-14.

For the US data, the 30-day case fatality rate is 13.5% (14.6% for women, 12.4% for men; mortality for women is higher because the age distribution for AMI is more heavily weighted towards older ages for women). For whites in the top 5% of counties, the case fatality rate is 12.4%; thus the ratio (or adjustment factor for whites in the highest-income US counties to the US average is the ratio, or 0.92.

We collect similar data from Denmark (2012-14) and Norway (2013-15) for the entire populations age 65+. In both countries, the sample was created using ICD10 codes I21.1-I21.4, and I21.9. In Norway, the case fatality rate (weighted by US population weights for age and sex)

© 2020 Emanuel EJ et al. *JAMA Internal Medicine*.

is 10.2% (11.7% for women, 8.8% for men), N = 20,496. The corresponding measures for Denmark is 10.7% (12.8% for women, 8.8% for men), N = 15,567.

2. OECD estimates and potential biases

We rely primarily on the publicly available OECD data age 45+ for the U.S. and 10 comparison high-income countries with available data for 2013-14. We are limited to 2013-14 in the OECD data because there is no more recent data available for U.S. 30-day mortality (case fatality) following the AMI. To calculate rates for the 45+ population in high-income counties, we apply the ratio 0.92 (derived above) to the overall OECD data for the U.S. That is, our estimate for the highest-income 5% of U.S. counties, 8.1, is equal to.92*8.8 per 100 AMI admissions; for the highest-income 1%, it is 8.4 (.95*8.8).

For OECD data, we combined two years of data and implemented confidence intervals reflecting both the reported annual confidence intervals and the two-year variability in rates across years. (We assumed a binary distribution for mortality at the individual level, which allowed us to recover sample sizes from each year's sample; this was then used to create confidence intervals for the combined two-year samples under the assumption that the samples in each year were independently drawn and equal in number).

As noted above, we used the linked hospitalization data to better capture true 30-day mortality, but there are more recent "unlinked" OECD data from 2016 that suggest a somewhat better ranking for the U.S. compared to other countries reporting data in that year. However, as has been pointed out by Drye et al. (2012), the link between in-hospital mortality and the corresponding 30-day mortality rate can be tenuous, most notably because differences in average length of stay differs so much across hospitals (as in Drye et al, 2012), or across countries. For

© 2020 Emanuel EJ et al. *JAMA Internal Medicine*.

example, even if 30-day mortality rates were identical in the U.S. and Great Britain, it is more likely that an AMI patient will die in hospital in Great Britain because average length of stay for AMI patients was 9.8 days compared to just 4.8 days in the U.S in 2010, the most recent data available (https://stats.oecd.org/Index.aspx?QueryId=51881).

Because there are no linked national databases for the general U.S. population between ages 45-64, the OECD used data from the Healthcare Cost and Utilization Project (HCUP) for a select group of states that are able to track readmissions from one hospital to another; one may then estimate case-fatality from either mortality occurring during the initial hospital admission, or during a readmission, but not if the death occurs outside of the hospital.

For this reason, U.S. case-fatality estimates are likely to be biased downward compared to other countries because they miss out-of-hospital deaths. For example, when we use the simple adjustment described above for the top 5% of counties to the U.S. average of 8.8%, the implied 30-day mortality rate is 8.1 percent, identical to the corresponding rate in Denmark. Yet as we have seen from the micro-level Danish (and Norwegian) universal data, mortality rates in the age 65+ population are consistently lower than U.S. mortality rates for higher-income counties and zip codes. If instead we calibrate not from the potentially biased U.S. data, we instead calibrate from the (e.g.) Danish data, we would predict an age 45+ mortality rate equal to 9.4% for the top 5% income counties, 9.6% for the top 1% of counties, and 9.0% for the top 5% of zip codes; all of these substantially larger than the median of comparison countries (8.2%). And while these estimates may themselves be biased upward because we are using fee-for-service Medicare claims data (rather than all enrollees including managed care patients), our alternative approach reinforces the earlier finding that even for privileged Americans, outcomes following AMI are no better than the median of the comparison countries.

© 2020 Emanuel EJ et al. *JAMA Internal Medicine*.

Citation:

Drye, E.E., Normand, S.L.T., Wang, Y., Ross, J.S., Schreiner, G.C., Han, L., Rapp, M. and Krumholz, H.M., 2012. Comparison of hospital risk-standardized mortality rates calculated by using in-hospital and 30-day models: an observational study with implications for hospital profiling. Annals of internal medicine, 156(1_Part_1), pp.19-26.

© 2020 Emanuel EJ et al. *JAMA Internal Medicine*.

eAppendix 2. List of 157 Highest-Incomes Counties in the US

State	County ID	State / County Name	Median Household Income in Dollars (2015)
51.00	51107	Loudoun County (VA)	125900.00
51.00	51610	Falls Church city (VA)	122092.00
51.00	51059	Fairfax County (VA)	112844.00
24.00	24027	Howard County (MD)	110224.00
8.00	8035	Douglas County (CO)	109926.00
35.00	35028	Los Alamos County (NM)	107126.00
47.00	47187	Williamson County (TN)	104367.00
51.00	51013	Arlington County (VA)	104354.00
34.00	34019	Hunterdon County (NJ)	102797.00
6.00	6085	Santa Clara County (CA)	102191.00
34.00	34027	Morris County (NJ)	101754.00
36.00	36059	Nassau County (NY)	101568.00
6.00	6081	San Mateo County (CA)	101133.00
6.00	6041	Marin County (CA)	99868.00
51.00	51600	Fairfax city (VA)	99671.00
51.00	51153	Prince William County (VA)	99206.00
34.00	34035	Somerset County (NJ)	99059.00
24.00	24009	Calvert County (MD)	98937.00
24.00	24031	Montgomery County (MD)	98314.00
13.00	13117	Forsyth County (GA)	97886.00
39.00	39041	Delaware County (OH)	97679.00
51.00	51179	Stafford County (VA)	95666.00
48.00	48157	Fort Bend County (TX)	95117.00
27.00	27019	Carver County (MN)	93857.00

© 2020 Emanuel EJ et al. *JAMA Internal Medicine*.

49.00	49043	Summit County (UT)	93235.00
25.00	25021	Norfolk County (MA)	93187.00
27.00	27139	Scott County (MN)	92898.00
48.00	48397	Rockwall County (TX)	92150.00
18.00	18057	Hamilton County (IN)	91844.00
24.00	24003	Anne Arundel County (MD)	90825.00
42.00	42029	Chester County (PA)	90555.00
6.00	6075	San Francisco County (CA)	90527.00
36.00	36079	Putnam County (NY)	90497.00
8.00	8039	Elbert County (CO)	90270.00
25.00	25017	Middlesex County (MA)	90025.00
38.00	38105	Williams County (ND)	89860.00
51.00	51061	Fauquier County (VA)	89610.00
51.00	51510	Alexandria city (VA)	89177.00
17.00	17093	Kendall County (IL)	88773.00
34.00	34003	Bergen County (NJ)	88512.00
27.00	27163	Washington County (MN)	88329.00
24.00	24017	Charles County (MD)	87941.00
36.00	36103	Suffolk County (NY)	87634.00
33.00	33015	Rockingham County (NH)	87103.00
48.00	48085	Collin County (TX)	86823.00
34.00	34025	Monmouth County (NJ)	86722.00
8.00	8014	Broomfield County (CO)	86548.00
9.00	9001	Fairfield County (CT)	86297.00
51.00	51075	Goochland County (VA)	86257.00
51.00	51735	Poquoson city (VA)	86135.00
25.00	25019	Nantucket County (MA)	86014.00
36.00	36119	Westchester County (NY)	85688.00
24.00	24013	Carroll County (MD)	84506.00

© 2020 Emanuel EJ et al. *JAMA Internal Medicine*.

34.00	34037	Sussex County (NJ)	84431.00
21.00	21185	Oldham County (KY)	84415.00
36.00	36087	Rockland County (NY)	84249.00
24.00	24035	Queen Anne's County (MD)	83914.00
24.00	24021	Frederick County (MD)	83746.00
56.00	56039	Teton County (WY)	83290.00
42.00	42091	Montgomery County (PA)	83258.00
24.00	24037	St. Mary's County (MD)	83148.00
56.00	56005	Campbell County (WY)	83042.00
6.00	6013	Contra Costa County (CA)	83036.00
20.00	20091	Johnson County (KS)	83007.00
51.00	51199	York County (VA)	83007.00
38.00	38053	McKenzie County (ND)	82906.00
2.00	2110	Juneau Borough (AK)	82892.00
9.00	9007	Middlesex County (CT)	82878.00
17.00	17097	Lake County (IL)	82160.00
51.00	51085	Hanover County (VA)	81900.00
53.00	53033	King County (WA)	81816.00
17.00	17043	DuPage County (IL)	81616.00
9.00	9013	Tolland County (CT)	81616.00
6.00	6001	Alameda County (CA)	81462.00
39.00	39165	Warren County (OH)	81383.00
19.00	19049	Dallas County (IA)	81381.00
49.00	49029	Morgan County (UT)	81358.00
51.00	51099	King George County (VA)	81128.00
2.00	2016	Aleutians West Census Area (AK)	80695.00
13.00	13113	Fayette County (GA)	80588.00
17.00	17111	McHenry County (IL)	80513.00
42.00	42017	Bucks County (PA)	80512.00

© 2020 Emanuel EJ et al. *JAMA Internal Medicine*.

55.00	55089	Ozaukee County (WI)	80135.00
48.00	48329	Midland County (TX)	79829.00
17.00	17133	Monroe County (IL)	79686.00
27.00	27141	Sherburne County (MN)	79495.00
51.00	51127	New Kent County (VA)	79322.00
6.00	6111	Ventura County (CA)	79285.00
48.00	48259	Kendall County (TX)	79108.00
55.00	55133	Waukesha County (WI)	78689.00
48.00	48491	Williamson County (TX)	78531.00
34.00	34023	Middlesex County (NJ)	78249.00
6.00	6059	Orange County (CA)	78002.00
24.00	24025	Harford County (MD)	77992.00
2.00	2020	Anchorage Borough (AK)	77791.00
51.00	51145	Powhatan County (VA)	77761.00
51.00	51095	James City County (VA)	77668.00
56.00	56035	Sublette County (WY)	77581.00
27.00	27037	Dakota County (MN)	77576.00
46.00	46083	Lincoln County (SD)	77540.00
48.00	48071	Chambers County (TX)	77282.00
26.00	26093	Livingston County (MI)	76934.00
38.00	38007	Billings County (ND)	76913.00
38.00	38089	Stark County (ND)	76844.00
34.00	34015	Gloucester County (NJ)	76780.00
32.00	32015	Lander County (NV)	76713.00
8.00	8097	Pitkin County (CO)	76663.00
2.00	2170	Matanuska-Susitna Borough (AK)	76601.00
15.00	15003	Honolulu County (HI)	76544.00
6.00	6069	San Benito County (CA)	76521.00
32.00	32007	Elko County (NV)	76518.00

© 2020 Emanuel EJ et al. *JAMA Internal Medicine*.

27.00	27171	Wright County (MN)	76407.00
13.00	13219	Oconee County (GA)	76371.00
24.00	24033	Prince George's County (MD)	76366.00
39.00	39055	Geauga County (OH)	76315.00
17.00	17197	Will County (IL)	76293.00
6.00	6061	Placer County (CA)	76203.00
51.00	51177	Spotsylvania County (VA)	76181.00
39.00	39159	Union County (OH)	76116.00
53.00	53061	Snohomish County (WA)	76053.00
48.00	48121	Denton County (TX)	75898.00
38.00	38057	Mercer County (ND)	75698.00
6.00	6017	El Dorado County (CA)	75575.00
51.00	51685	Manassas Park city (VA)	75429.00
44.00	44001	Bristol County (RI)	75324.00
13.00	13073	Columbia County (GA)	75232.00
8.00	8037	Eagle County (CO)	75191.00
18.00	18011	Boone County (IN)	75163.00
36.00	36061	New York County (NY)	75136.00
49.00	49051	Wasatch County (UT)	75112.00
51.00	51041	Chesterfield County (VA)	75107.00
36.00	36091	Saratoga County (NY)	75029.00
13.00	13057	Cherokee County (GA)	74885.00
48.00	48173	Glasscock County (TX)	74854.00
22.00	22005	Ascension Parish (LA)	74852.00
34.00	34005	Burlington County (NJ)	74844.00
25.00	25023	Plymouth County (MA)	74736.00
55.00	55109	St. Croix County (WI)	74624.00
46.00	46127	Union County (SD)	74439.00
29.00	29183	St. Charles County (MO)	74009.00

© 2020 Emanuel EJ et al. *JAMA Internal Medicine*.

31.00	31153	Sarpy County (NE)	73856.00
18.00	18063	Hendricks County (IN)	73478.00
33.00	33011	Hillsborough County (NH)	73474.00
27.00	27003	Anoka County (MN)	73276.00
38.00	38025	Dunn County (ND)	73135.00
11.00	11001	District of Columbia (DC)	73115.00
2.00	2068	Denali Borough (AK)	73000.00
6.00	6055	Napa County (CA)	72683.00
51.00	51683	Manassas city (VA)	72562.00
29.00	29165	Platte County (MO)	72548.00
48.00	48339	Montgomery County (TX)	72428.00
8.00	8013	Boulder County (CO)	72392.00
49.00	49011	Davis County (UT)	72268.00
48.00	48003	Andrews County (TX)	72184.00
34.00	34021	Mercer County (NJ)	72172.00
44.00	44009	Washington County (RI)	71951.00
17.00	17063	Grundy County (IL)	71928.00

© 2020 Emanuel EJ et al. *JAMA Internal Medicine*.
